# Comparative Effectiveness of Exercise Training for Patients With Chronic Thromboembolic Pulmonary Hypertension After Pulmonary Endarterectomy: A Systematic Review and Meta-Analysis

**DOI:** 10.3389/fcvm.2021.664984

**Published:** 2021-06-17

**Authors:** Ya-Lin Zhao, Ping Yuan, Qin-Hua Zhao, Su-Gang Gong, Rui Zhang, Jing He, Ci-Jun Luo, Hong-Ling Qiu, Jin-Ming Liu, Lan Wang, Rong Jiang

**Affiliations:** ^1^Department of Respiratory Critical Care Medicine, The First Hospital of Kunming, Kunming, China; ^2^Department of Cardio-Pulmonary Circulation, Shanghai Pulmonary Hospital, Tongji University School of Medicine, Shanghai, China

**Keywords:** pulmonary endarterectomy, pulmonary hypertension, exercise intolerance, cardiorespiratory fitness, exercise training

## Abstract

**Background:** Patients with chronic thromboembolic pulmonary hypertension (CTEPH) still experience reduced exercise capacity despite pulmonary endarterectomy (PEA). Exercise training improves the exercise capacity and quality of life (QoL) in patients with PH, but data on the effects of exercise training on these patients are scarce. The aim of this meta-analysis and systematic review was to evaluate the effectiveness and safety of exercise training in CTEPH after PEA.

**Methods:** We searched the relevant literature published before January 2020 for the systematic review and meta-analysis using the PubMed, EMBASE, and Cochrane Library databases. The primary outcome was a change in the 6-min walking distance (6 MWD). We also assessed the effect of exercise on the peak oxygen uptake (VO_2_) or peak VO_2_/kg, oxygen uptake anaerobic threshold, workload, oxygen pulse, hemodynamics, arterial blood gases, oxygen saturation, N-terminal pro-brain-type natriuretic peptide (NT-proBNP), quality of life (QoL) and pulmonary function tests.

**Results:** We included 4 studies with 208 exercise-training participants. In the pooled analysis, short-term exercise training can improve the 6 MWD of 58.89 m (95% CI: 46.26–71.52 m, *P* < 0.0001). There was a significant increase in the peak VO_2_/kg or peak VO_2_ after exercise training (3.15 ml/min/kg, 95% CI: 0.82–5.48, *P* = 0.008; 292.69 ml/min, 95% CI: 24.62–560.75, *P* = 0.032, respectively). After exercise training, the maximal workload and O_2_ pulse significantly improved. Three months of exercise training increased the right ventricular ejection fraction by 3.53% (95% CI: 6.31–11.94, *P* < 0.00001, *I*^2^ = 0) independently of PEA surgery. In addition, NT-proBNP plasma levels significantly improved with exercise training after PEA [weighted mean difference (WMD): −524.79 ng/L, 95% CI: 705.16 to −344.42, *P* < 0.0001, *I*^2^ = 0]. The partial pressure of oxygen and pH improved progressively over 12 weeks of exercise training (WMD: 4 mmHg, 95% CI: 1.01–8.33, *P* = 0.01; WMD: 0.03, 95% CI: 0.02–0.04, *P* < 0.0001, respectively). Subscales of the QoL measured by the SF-36 questionnaire had also improved. In addition, exercise training was well-tolerated with a low dropout rate, and no major adverse events occurred during exercise training.

**Conclusion:** Exercise training may be associated with a significant improvement in the exercise capacity and QoL among CTEPH patients after PEA and was proven to be safe. However, more large-scale multicentre studies are needed to confirm the effectiveness and safety of exercise training in CTEPH patients after PEA.

**PROSPERO registration number:** CRD42021235275.

## Introduction

Chronic thromboembolic pulmonary hypertension (CTEPH) is characterized by stenosis and/or occlusion of pulmonary arteries caused by organized thrombus (1). Early diagnosis and suitable treatment are critical because CTEPH has a high rate of mortality and right-side heart failure (2). Pulmonary endarterectomy (PEA) is a proven curative treatment for operable CTEPH (3, 4). PEA surgery is recommended as the first-line therapy for operable CTEPH patients (ClassI, evidence C) (5, 6). However, some patients still suffer limited exercise capacity despite PEA (7), especially when their pulmonary hemodynamics do not normalize (8, 9). An improvement in exercise tolerance, physical function, and the ability to cope with daily living should be targets during the comprehensive management of these patients (10).

Rehabilitation programmes, including aerobic exercise training, have strong evidence of effectiveness in improving the exercise capacity, dyspnoea, and health-related quality of life (QoL) in different etiologies of pulmonary hypertension (PH) (11–17). Exercise training has also improved muscular and right ventricular function, QoL and pulmonary hemodynamics in idiopathic pulmonary arterial hypertension (PAH), PAH associated with connective tissue diseases and inoperable CTEPH (11–16, 18–20).

Exercise training has improved the exercise capacity for up to 3 months in patients with CTEPH after PEA, independent of the post-surgery hemodynamic response (17, 21–23). However, the evidence of the effect of exercise training on patients with CTEPH after PEA is limited.

Therefore, we performed the meta-analysis and systematic review to assess the efficacy and safety of an exercise training program in CTEPH patients after PEA.

## Methods and Analysis

### Data Sources and Search Strategy

We designed the study according to the PRISMA statement (Supplementary Table 1) (24). We searched the PubMed, EMBASE, and the Cochrane Collaboration databases using the key words “exercise training,” “chronic thromboembolic pulmonary hypertension,” “pulmonary endarterectomy,” and “rehabilitation” to identify studies that evaluated the efficacy and safety of exercise training for CTEPH patients after PEA surgery. Our search included articles published from the database beginning up to January 2020. The search was limited to human studies and English language articles.

### Study Selection

We included five observational studies comparing the effectiveness of exercise training in CTEPH patients after PEA. The inclusion criteria were (1) patients were diagnosed with CTEPH and (2) the patients underwent exercise training after PEA. We excluded studies if they were conference abstracts, case reports, reviews, letters, or editorials.

### Data Extraction

Two independent reviewers (Y-L. Z, R.J) performed the literature search, data extraction, and methodological grading. Disagreements were resolved by consensus. We extracted the information of each study, such as the author, year of publication, demographic characteristics, nature of the study, hemodynamics, and pre- and post-exercise intervention results.

### Outcomes

The primary outcome was a change in the 6-min walking distance (6 MWD). The secondary outcomes were as follows:

1. Changes in exercise tolerance by cardiopulmonary exercise testing (CPET): oxygen consumption at peak exercise (peak VO_2_ and peak VO_2_/kg), oxygen uptake anaerobic threshold (VO_2_ at AT), and workload;
2. Changes in cardiac function by CPET: oxygen pulse, resting and peak heart rate (HR), and resting and peak oxygen saturation;
3. Changes in pulmonary hemodynamics by right heart catheterization: right atrial pressure (RAP), mean pulmonary artery pressure (mPAP), pulmonary vascular resistance (PVR), pulmonary capillary wedge pressure (PCWP), transpulmonary gradient (TPG), systemic vascular resistance (SVR), total pulmonary resistance (TPR), cardiac output (CO), cardiac index (CI) and right ventricular ejection fraction (RVEF).
4. Changes in the resting and peak exercise systolic pulmonary arterial pressure (sPAP) by echocardiography;
5. Changes in N-terminal pro-brain-type natriuretic peptide (NT-proBNP);
6. Changes in arterial blood gases: pH, oxygen partial pressure (PaO_2_), and partial pressure of carbon dioxide (PaCO_2_);
7. Changes in QoL scales assessed by the SF-36 questionnaire;
8. Changes in echocardiography, including the resting and peak systolic pulmonary pressure (sPAP);
9. Changes in pulmonary function tests: forced vital capacity (FVC) and forced expiratory volume at 1 second (FEV1).

### Methodological Quality

We used the NIH quality assessment tool to assess the quality of pre-post interventional studies (25). Due to the insufficient number of research articles, publication bias could not be assessed.

### Data Synthesis and Statistical Analysis

We conducted meta-analyses for comparisons when two or more studies reported the same outcome. Continuous values are presented as the mean ± SD and analyzed using weighted mean differences (WMDs). We used random-effect models to quantitatively synthesize the evidence and to calculate the summary estimates. Pooled analysis was calculated using fixed-effect models, and random-effect models were applied in cases of significant heterogeneity.

Certain studies reported continuous variables in the form of interquartile ranges or 95% confidence intervals (CI) other than the SD and needed to be converted into SDs. Cases in which converted SDs could not be obtained were excluded. We considered *P* < 0.05 as significant.

Statistical analysis was performed using Stata version 15 software (Stata Corp., College Station, Texas) and RevMan 5.4 (The Cochrane Collaboration, Copenhagen, Oxford, UK).

## Results

### Characteristics of the Studies

**Figure 1:**
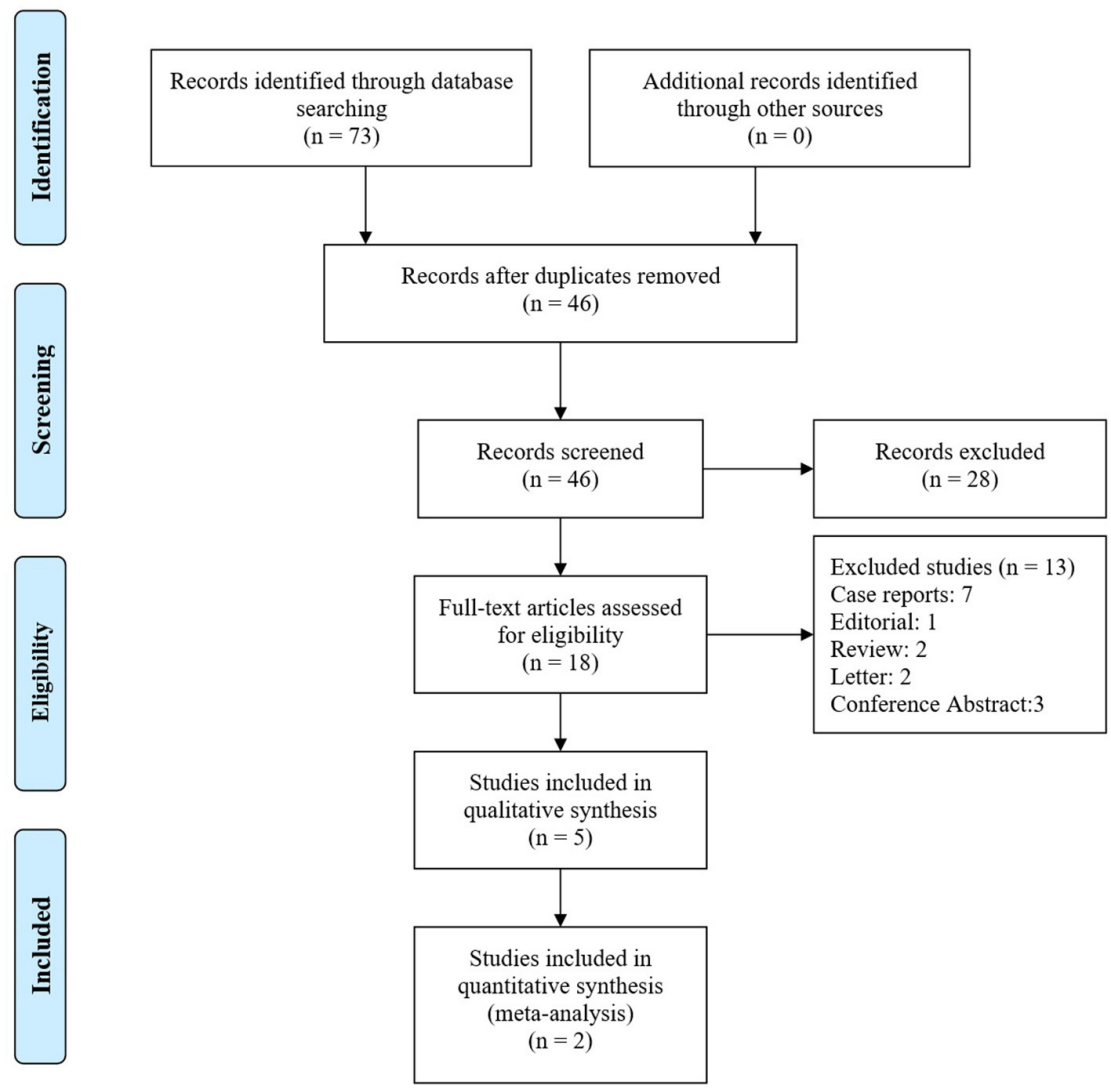
Flow diagram.

We retrieved 73 articles for more detailed analysis after 68 initial articles were identified by the search and included 4 studies in our systematic review (Figure 1). There were four pre-post interventional studies, encompassing 208 exercise-training participants between 2012 and 2020 (17, 21–23). All studies used a supervised exercise training programme combined with aerobic exercise (treadmill or bicycle Ergometer) and resistance training. One study was performed at an outpatient rehabilitation center, while three studies were performed in-hospital for the first few weeks followed by home-based exercise training. All training participants underwent low workload aerobic exercise training with some form of resistance and respiratory training. The exercise intensity was titrated at 50–70% of the peak exercise capacity. The characteristics of all included studies are presented in Table 1.

**Table 1:** Characteristics of all included studies.

**References**	**No. of patients**	**Exercise training intervention**	**Duration and time interval between PEA and the exercise programme**	**Primary endpoint**	**Results**
Nagel et al. (17)	*n* = 45; Female:16; Age: 61 ± 15 years	• In the Rehabilitation Clinic (first 3 weeks): Interval bicycle ergometer, walking, respiratory training (low workloads, 5 days/week, a minimum of 1.5 h/day), single muscle groups (low weights). • Training at home (for 12 weeks): Bicycle ergometer (≥ 30 min/day at 5 days a week). • Psychological support and mental training.	3 weeks; 15 weeks. Time interval: not described	6 MWD; CPET variables; stress Doppler echocardiography; HR; blood pressure; Borg dyspnoea index; WHO-FC; SF-36; NT-proBNP; gas exchange	6 MWD↑; SF-36 scores for physical functioning and vitality↑; peak VO_2_↑; peak VO_2_/kg↑; workload↑; an increase of maximal HR↑; NT-proBNP↓; WHO-FC(-).
Inagaki et al. (23)	*n* = 8; Age: 64 ± 12 years	In-hospital each week (40–60 min) and home-based programme, including: • Lower-limb endurance training (walking exercises, a cycle ergometer at 60% of the target HR) • Lower and upper limb strength training • Respiratory exercises • Education	12 weeks Time interval: not described	Echocardiography; BNP; exercise capacity; dyspnoea severity and the functional status; pulmonary function; peripheral muscle force; physical activity.	6 MWD↑; TDI scores↑; QF↑; Ex↑; SGRQ scores↑; No change in MRC scores, BDI scores, HRR1, and WHO-FC.
La Rovere et al. (21)	Group 1 = 84, Age: 60.4 ± 13.8 years, Female: 6; Group 2 = 26, Age: 57.9 ± 13.1 years, Female: 31.	Daily sessions of: • Incremental exercise training (30 min, at 50–70% of the maximal load); • Abdominal, upper, and lower limb muscle activities including lifting progressively increasing light weights (0.30–0.50 kg), and shoulder and full arm circling; • Education; • Nutritional programmes and psychosocial counseling.	3 months Time interval: not described	6 MWD; pulse oximetric oxygen saturation, lung function tests; arterial blood gases; hemodynamics.	PAP↓; TPG↓; RVEF↑; PVR↓; PaO2↑; pH↓; No changes in RAP, PCWP, CO, CI, SVR, PVR,TPR, PaCO2, FEV1,% predicted, FVC, % predicted, and FEV1/FVC, % predicted^*^.
Nagel et al. (22)	*n* = 45; Female: 22 Age: 57.6 ± 12.4 years.	• In the rehabilitation clinic (first 3 weeks): Interval bicycle ergometer, walking, respiratory training (low workloads, 5 days/week, a minimum of 1.5 h/day), single muscle groups (low weights). • Training at home (for 19 weeks): Bicycle ergometer (≥ 15 min/day at 5 days a week). • Psychological support and mental training	3 weeks; 19 weeks. Time interval: 3.3 ± 0.9 (median 3.1) weeks	WHO-FC; 6 MWD; BDI scores; echocardiography; lung function; blood gas; SF-36; CPET with stress echocardiography.	6 MWD↑; SF-36 scores for physical functioning and vitality↑; peak VO_2_↑; peak VO_2_/kg↑; workload↑; NT-proBNP↓; RA area↓; RV area↓; sPAP↓; TAPSE↑; left ventricular eccentricity index↓; tissue Doppler imaging s RV free wall↑; oxygen pulse↑; EqCO_2_↓; BDI↓; HR↓; peak HR↓; O_2_ at AT↑.

*VE, ventilation; VO_2_, oxygen consumption; VCO2, carbon dioxide output; WHO-FC, WHO functional class; NT-proBNP, N-terminal pro-brain natriuretic peptide; 6 MWD, 6-minute walking distance; TRPG, tricuspid regurgitation pressure gradient; sPAP, systolic pulmonary arterial pressure; BNP, brain natriuretic peptide; MRC, Medical Research Council dyspnoea grade; BDI, dyspnoea index; TDI, Transition dyspnoea index. QF, quadriceps force; HF, handgrip force; HHR1, heart rate recovery during the first minute; HR, heart rate; NRADL, Nagasaki University Respiratory ADL Questionnaire; SGRQ, St. George's Respiratory Questionnaire; Ex, amount of exercise; PAP, pulmonary artery pressure; PCWP, pulmonary capillary wedge pressure; TPG, transpulmonary gradient; CO, cardiac output; RVEF, right ventricular ejection fraction; SVR, systemic vascular resistance; PVR, pulmonary vascular resistance; TPR, transpulmonary resistance; FVC, forced vital capacity; FEV1, forced expiratory volume at 1 second; RA, right atrial; RV, right ventricular; TAPSE, tricuspid annular plane systolic excursion; SaO_2_, oxygen saturation; EqCO_2_:respiratory equivalent for CO_2_*.

* *COMPARED integrated two groups with 3-month vs. after surgery (before rehabilitation)*.

### Quality Assessment

The quality assessment of the pre-post interventional studies had a detailed description. All included studies had a clearly stated study question, prespecified eligibility/selection criteria, CTEPH patient's representative in the real world, clearly defined intervention and outcome variables, and low rate of loss to follow-up (Supplemental Table 2).

### Six-Minute Walking Distances

**Figure 2:**
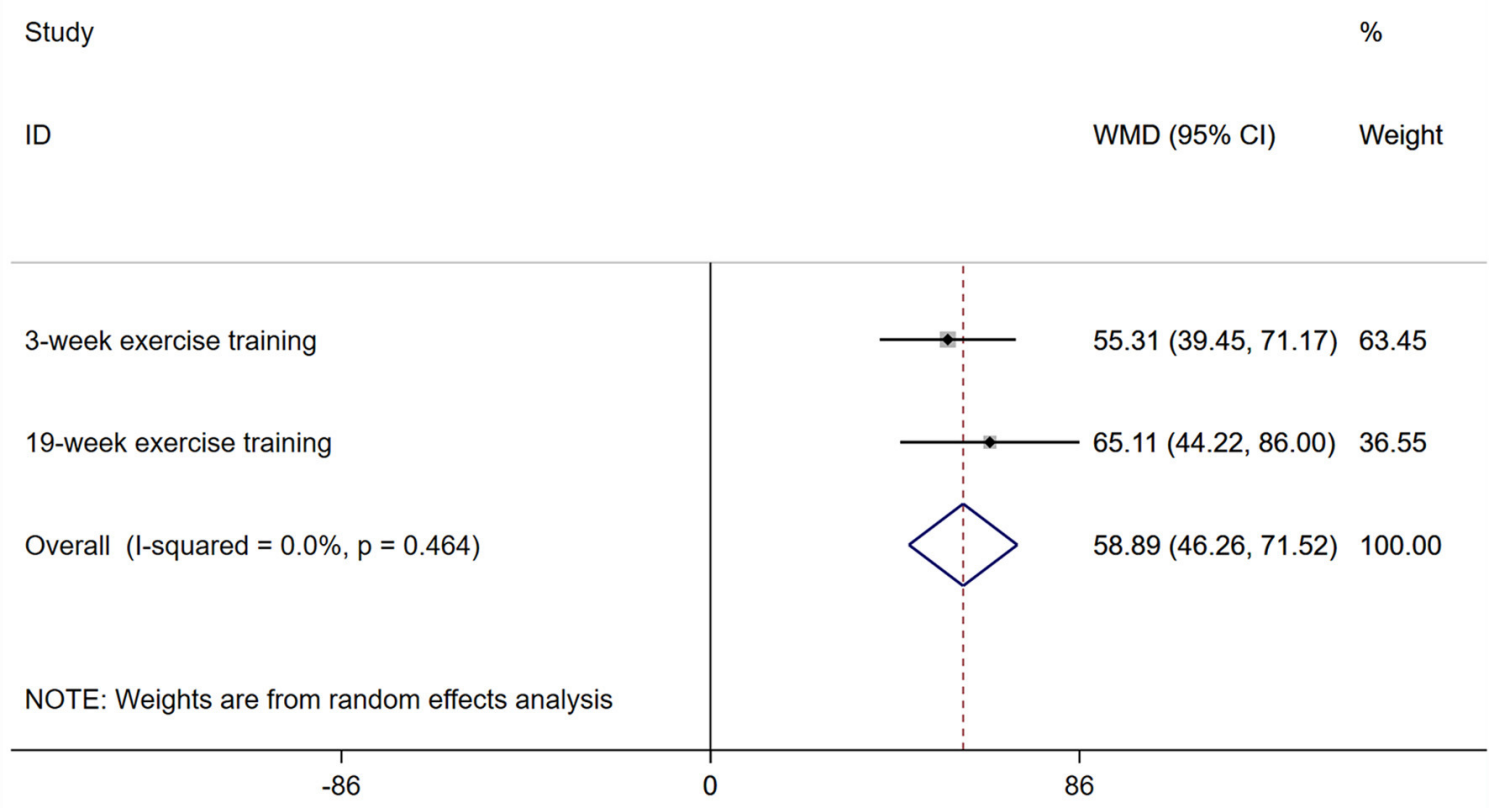
Forest plots for the 6-min walking distance. 6MWD, six-minute walking distance.

In Christian Nagel's study (22), the 6 MWD significantly improved by 55.31 ± 53.67 m (95% CI: 39.19–71.43, *P* < 0.0001) and 65.11 ± 63.96 m (95% CI: 43.14–87.09, *P* < 0.0001) with 3- or 19-week exercise training after PEA in patients with CTEPH, respectively. By pooling analysis, short-term exercise training can improve the 6 MWD of 58.89 m (95% CI: 46.26–71.52 m, *P* < 0.0001) (Figure 2). Ekkehard Grünig evaluated the effects of exercise training in patients with inoperable or residual CTEPH in a prospective study (17). The 6 MWD significantly improved by 71 ± 70 m after 15 weeks among 35 inoperable or residual CTEPH patients (*P* = 0.001). Jiro Terada also evaluated the changes in the 6 MWD after a 12-week exercise training programme in 8 inoperable or residual CTEPH patients (23). After completion of the pulmonary rehabilitation programme, the 6 MWD significantly improved by 33.3 ± 25.1 m compared with the baseline (*P* < 0.01). However, we could not extract information on residual CTEPH from the studies of Ekkehard Grünig and Jiro Terada.

### Cardiopulmonary Exercise Testing

Patients performed a gradually increasing work rate CPET to maximal tolerance on an electromagnetically braked cycle ergometer in the upright position. The peak VO_2_, peak VO_2_/kg, VO_2_ at AT, peak workload, oxygen pulse, HR and oxygen saturation were evaluated to assess the CPET capacity.

Regarding exercise tolerance, Christian Nagel's study (22) was included to analyse the peak VO_2_, peak VO_2_/kg, VO_2_ at AT and peak workload by pooling analysis.

There was a significant increase in the peak VO_2_/kg or peak VO_2_ after exercise training (3.15 ml/min/kg, 95% CI: 0.82–5.48, *P* = 0.008; 292.69 ml/min, 95% CI: 24.62–560.75, *P* = 0.032, respectively). However, there were no significant increases in VO_2_ at AT (136.32 ml/min, 95% CI: −66.78–339.41, *P* = 0.19). After exercise training, the max workload during exercise had significantly improved (26.69 Watt, 95% CI: 9.41–43.98, *P* = 0.002) (Table 2).

**Table 2:** Changes in cardiopulmonary exercise testing after exercise training in CTEPH patients with PEA.

**Variable (22)**	**WMD (95% CI)**	**% Weight**	***P*-value**
**Exercise tolerance**			
Peak VO_2_/kg	3.15 (0.82, 5.48)	53.78	0.008
after 3 weeks	1.99 (1.48, 2.50)	16.26	
after 12/15 weeks	4.37 (3.48, 5.26)	16.17	
Peak VO_2_	292.69 (24.62, 560.75)	3.45	0.032
after 3 weeks	158.50 (122.50,194.50)	1.08	
after 12/15 weeks	432.09 (349.76, 514.42)	0.22	
VO_2_ at AT	136.32 (−66.78, 339.41)	1.11	0.188
after 3 weeks	37.07 (−34.72, 108.86)	0.29	
after 12/15 weeks	244.50 (133.71, 355.29)	0.12	
Workload max	26.69 (9.41, 43.98)	41.65	0.002
after 3 weeks	18.18 (13.05, 23.31)	12.69	
after 12/15 weeks	35.83 (27.53, 44.13)	9.33	
**Cardiac function**			
O_2_ pulse	1.55 (0.40, 2.70)	33.09	0.008
after 3 weeks	0.98 (0.48, 1.48)	11.41	
after 12/15 weeks	2.15 (1.50, 2.80)	10.97	
HR rest	−2.70 (−7.75, 2.35)	10.8	0.295
after 3 weeks	−0.36 (−3.16, 2.44)	4.09	
after 12/15 weeks	−5.54 (−9.76, −1.32)	2.21	
HR max	10.41 (−0.66, 21.48)	4.66	0.065
after 3 weeks	4.95 (−0.20, 10.10)	1.58	
after12/15 weeks	16.25 (9.69, 22.81)	1.03	
SaO_2_ rest	0.55 (0.02, 1.08)	31.10	0.043
after 3 weeks	0.39 (−0.24, 1.02)	11.02	
after 12/15 weeks	0.94 (−0.05, 1.93)	9.72	
SaO_2_ max	1.18 (−0.18, 2.54)	20.35	0.088
after 3 weeks	0.57 (−1.31, 2.45)	6.41	
after 12/15weeks	1.85 (−0.11, 3.81)	6.16	

*SaO_2_, oxygen saturation; PEA, pulmonary endarterectomy; HR, heart rate; Oxygen consumption, O_2_; WMD, weighted mean difference*.

With regard to cardiac function, we pooled the study of Christian Nagel to analyse the oxygen pulse, resting and peak HR, and resting and peak oxygen saturation (22). By exercise training, the O_2_ pulse and oxygen saturation had significantly improved (1.55, 95% CI: 0.40–2.70, *P* = 0.008; 0.55%, 95% CI: 0.02–1.08, *P* = 0.043), with a tread of an improved of maximal HR of 10.41 bpm (0.66, 95% CI: −0.66 to 21.48, *P* = 0.065) and maximal saturation of 1.18% (95% CI: −0.18 to 2.54, *P* = 0.088) during exercise (Table 2). In Ekkehard Grünig's study (17), among patients with inoperable or residual CTEPH after PEA, the peak oxygen consumption, maximal workload and maximal HR had improved (Supplementary Table 3).

### Effect of PEA on Hemodynamic Measurements

In Nicolino Ambrosino's study (21), CTEPH patients were divided into Group 1 (*n* = 84) and Group 2 (*n* = 26) according to the post-surgery hemodynamic response. Group 1 patients met at least one of the following criteria: (1) mPAP ≤ 25 mm Hg; (2) ≥50% reduction in mPAP; and (3) ≥70% reduction in PVR. Group 2 included patients who did not meet any of these criteria. In this study, we combined the mean and SD of the two groups before PEA, after PEA and after exercise training.

CTEPH patients had a decreased mPAP of 18.10 mmHg, PCWP of 1.46 mmHg, TPG of 20.01 mmHg, SVR of 548.87 dynes/cm^−5^, PVR of 528.61 dynes/cm^−5^, and TPR of 532 dynes/cm^−5^ after PEA surgery. PEA surgery increased the CO, CI and RVEF. However, the RAP remained unchanged (Table 3).

**Table 3:** Forest plots of pulmonary hemodynamic measurements.

**Variables (21)**	**Pulmonary hemodynamic measurements after PEA before rehabilitation**		**Pulmonary hemodynamic measurements with 3 months of exercise training after PEA**	
	**MD (95% CI)**	***P*-value**	**MD (95% CI)**	***P*-value**
RAP, mmHg	−0.04 (−2.26, 2.18)	0.97	−0.14 (−0.94, 0.66)	0.74
mPAP, mmHg	−18.10 (−29.35, −6.84)	0.002	−0.18 (−5.60, 5.25)	0.95
PCWP, mmHg	1.46 (0.04, 2.87)	0.04	−0.24 (−1.10, 0.63)	0.59
TPG, mmHg	−20.01 (−29.98, −10.03)	<0.0001	0.34 (−4.36, 5.05)	0.89
CO, L/min	0.92 (0.59, 1.25)	<0.00001	0.20 (−0.10, 0.50)	0.19
CI, L/min/m^2^	0.49 (0.20, 0.77)	0.0009	0.10 (−0.04, 0.24)	0.17
RVEF, %	9.12 (6.31, 11.94)	<0.00001	3.53 (1.11, 5.95)	0.004
SVR, dyn·s·cm^−5^	−548.87 (−687.29, −410.44)	<0.00001	92.20 (−15.24, 199.63)	0.09
PVR, dyn·s·cm^−5^	−538.61 (−744.64, −332.59)	<0.00001	1.19 (−78.37, 80.75)	0.98
TPR, dyn·s·cm^−5^	−532.00 (−773.86, −290.13)	<0.0001	−10.72 (−103.32, 81.87)	0.82

*RAP, right atrial pressure; mPAP, mean pulmonary arterial pressure; PCWP, pulmonary capillary wedge pressure; TPG, transpulmonary gradient; CO, cardiac output; CI, cardiac index; RVEF, right ventricular ejection fraction; SVR, systemic vascular resistance; PVR, pulmonary vascular resistance; TPR, transpulmonary resistance; PEA, pulmonary endarterectomy; MD, mean difference*.

### Effect of Exercise Training After PEA on Hemodynamic Measurements

Table 3 shows the changes in hemodynamics from immediately after the surgery (before rehabilitation) to 3 months after the exercise training. Three months of exercise training increased the RVEF by 3.53% (95% CI: 6.31–11.94, *P* < 0.00001, *I*^2^ = 0). However, 3 months of exercise training did not influence the RAP, mPAP, PCWP, TPG, CO, CI, SVR, PVR or TPR (Table 3).

### NT-proBNP, Arterial Blood Gases, Echocardiography, and Pulmonary Function Test

By pooling analysis in Christian Nagel's study (22), we found that the NT-proBNP plasma levels had continuously decreased by 524.79 ng/L (95% CI: −705.16 to −344.42, *P* < 0.0001, *I*^2^ = 0) when CTEPH patients underwent 3–19 weeks of exercise training after PEA surgery (Table 4).

**Table 4:** The effect of exercise training on NT-proBNP, arterial blood gases, echocardiography, and pulmonary function test in patients with CTEPH after PEA.

**Variables**	**WMD (95% CI)**	***I*^**2**^ (%)**	***P*-value**
**NT-proBNP, ng/L** (22)	−524.79 (−705.16, −344.42)	0	<0.0001
Changes after 3 weeks	−482.30 (−698.90, −269.70)		
Changes after 19 weeks	−622.21 (−955.09, −289.33)		
**Arterial blood gases** **(****21****)**			
**From before surgery to after surgery (before rehabilitation)**			
PaO_2_, mmHg	9.30 (6.04, 12.55)	0	<0.00001
PaCO_2_, mmHg	1.72 (0.54, 2.89)	48	0.004
pH	0.01 (0, 0.02)	0	0.04
**From after surgery (before rehabilitation) to 3 months rehabilitation**			
PaO_2_, mmHg	4.67 (1.01, 8.33)	0	0.01
PaCO_2_, mmHg	0.90 (−0.28, 2.08)	0	0.13
pH	−0.03 (−0.04, −0.02)	0	<0.00001
**Echocardiography** **(****22****)**			
After 3 weeks	−1.20 (−3.14, 0.74)	0	0.225
sPAP rest	−1.29 (−3.55, 0.97)		
sPAP max	−0.95 (−4.74, 2.84)		
After 19 weeks	7.42 (3.87, 10.97)	0	<0.0001
sPAP rest	6.37 (2.23, 10.51)		
sPAP max	10.35 (3.44, 17.26)		
**Pulmonary function test** **(****21****)**			
FVC % predicted	4.10 (−0.63, 8.83)	0	0.09
FEV1 % predicted	3.30 (−2.72, 9.32)	0	0.28
FEV1/FVC % predicted	2.70 (−1.13, 6.53)	0	0.17

*NT-proBNP, N-terminal pro-brain-type natriuretic peptide; PEA, pulmonary endarterectomy; PaO_2_, oxygen partial pressure; PaCO_2_, partial pressure of carbon dioxide; sPAP, systolic pulmonary arterial pressure; FVC, forced vital capacity; FEV1, forced expiratory volume at 1 second; WMD, weighted mean difference*.

By combining the mean and SD of the two groups in Nicolino Ambrosino's study (21), PEA surgery significantly improved the PaO_2_ by 9.30 mmHg, PaCO_2_ by 1.72 mmHg and pH by 0.01 (Table 4). Three months of exercise training after PEA surgery further improved the PaO_2_ and the pH (WMD: 4 mmHg, 95% CI: 1.01–8.33, *P* = 0.01; WMD: 0.03, 95% CI: 0.02–0.04, *P* < 0.0001, respectively), but not the PaCO_2_ (Table 4).

In Christian Nagel's study (22), the resting sPAP or maximal sPAP during exercise training improved with the extension of rehabilitation (Table 4). Ekkehard Grünig's study (17) and Jiro Terada's study (23) also assessed the sPAP by echocardiography. In the two interventional studies (17, 23), there were no significant improvements in resting sPAP or maximal sPAP. We cannot conduct quantitative meta-analysis of the sPAP in the above three studies because Ekkehard Grünig's and Jiro Terada's studies included some inoperable CTEPH patients.

In addition, the right atrial and right ventricular areas, tricuspid annular plane systolic excursion (TAPSE), left ventricular eccentricity index, pulmonary artery diameter, and tissue Doppler imaging of the RV free wall, as assessed by echocardiography, improved by varying degrees with exercise training (22).

In Christian Nagel's study (22), neither the FVC% predicted, FEV1% predicted nor FEV1/FVC% predicted improved after 12 weeks of exercise training after PEA.

### Quality of Life

**Figure 3:**
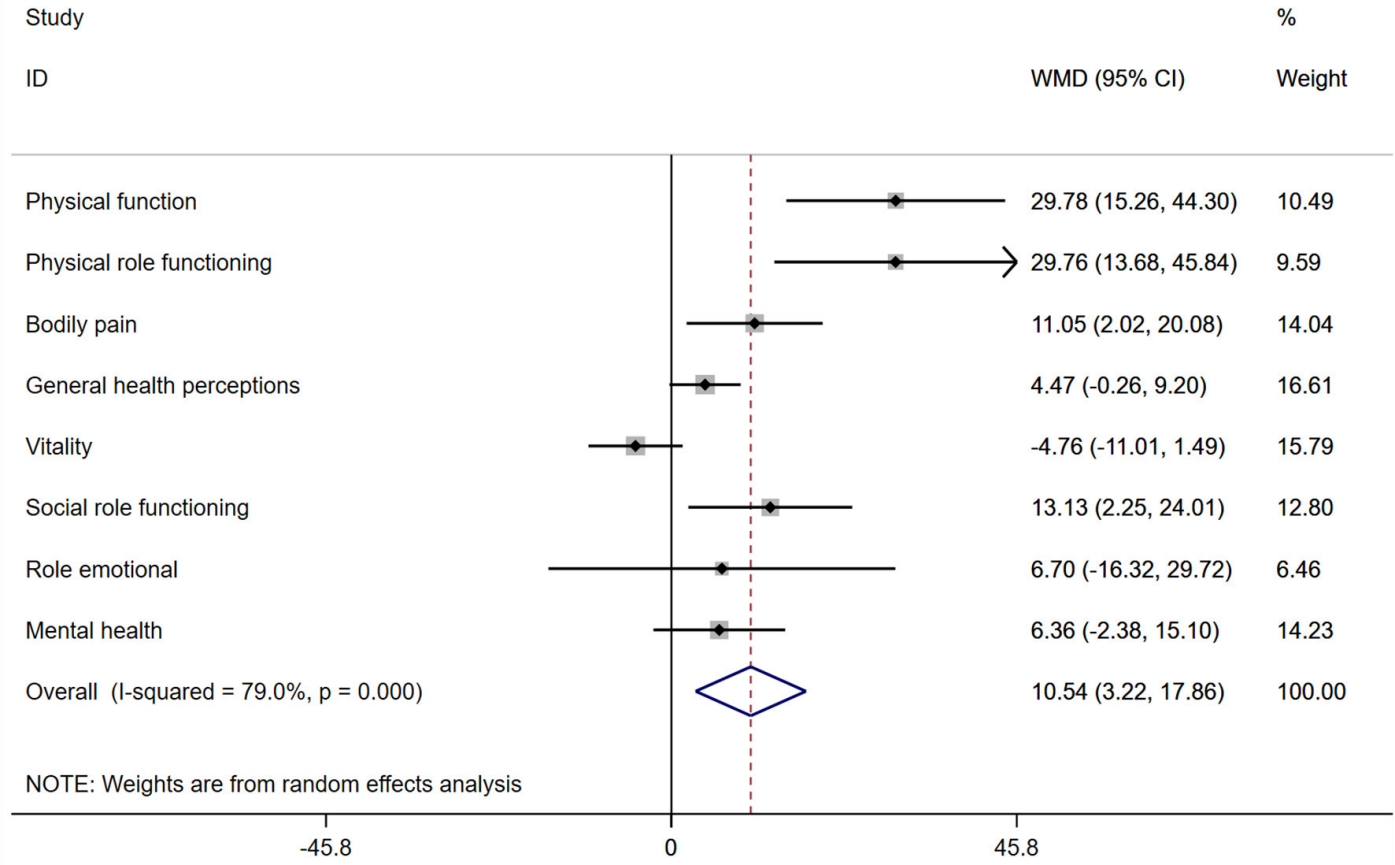
Forest plots for the quality of life. IV, inverse variance.

Christian Nagel's study described the QoL change after exercise training in CTEPH patients after PEA (22). The subscales for physical function (WMD: 29.78 points, 95% CI: 15.26–44.30, *P* < 0.0001), physical role functioning (WMD: 29.76 points, 95% CI: 13.68–45.84, *P* < 0.0001), bodily pain (WMD: 11.05 points, 95% CI: 2.02–20.08, *P* = 0.017), and social role functioning (WMD: 13.13 points, 95% CI: 2.25–24.01, *P* = 0.018) improved during the 19-week exercise training after PEA (Figure 3). However, physical function, general health perception, physical role functioning, vitality, emotional role and mental health had not significantly improved (Figure 3).

### Safety of Exercise Training

In most of the included studies, exercise training was well-tolerated with an overall dropout rate of 5%. Approximately 0.9% of the training patients experienced syncope or palpitations, half of which were related to exercise training (2.2%). Furthermore, during exercise training, no major adverse events, such as symptom progression, right heart failure, or death, occurred among participants (Table 5).

**Table 5:** Adverse effects reported in the included studies.

**References**	**Total no. of exercise training participants**	**Exercise training related adverse events**
Nagel et al. (17)	45	Syncope in one patient; Herpes zoster infection in one patient.
Shamseer et al. (24)	8	Low blood pressure and tachycardia in one patient
La Rovere et al. (21)	110	None
Nagel et al. (22)	45	None

## Discussion

The principal finding of our study is that exercise training significantly improves exercise capacity and cardiorespiratory fitness among CTEPH patients after PEA. There was also a significant reduction in the NT-proBNP and an improvement in the arterial blood gases. Furthermore, exercise training was well-tolerated with significant improvements in the QoL.

Our study has important clinical implications. Exercise rehabilitation has been actively discouraged in PAH patients because of the fear that it would worsen symptoms and negatively impact cardiac function. However, recently, with evidence of exercise rehabilitation in PH, supervised rehabilitation, including exercise training, has been recommended for patients with PH (Class I,A) (26). The 2019 ERS statement on exercise training and rehabilitation acknowledges the strong evidence of benefits from exercise training in PH (27). Exercise training has shown beneficial effects as an add-on to PAH-specific drug therapies among CTEPH patients (17). Current guidelines recommend PEA as a potentially curative first-choice treatment that is superior to medical therapies in inoperable CTEPH patients (28, 29). Our study findings provide comprehensive evidence to support the efficacy and safety of exercise training in patients with CTEPH after PEA.

In Angelo G. Corsico's study, most of the CTEPH patients recovered good exercise tolerance (30). However, ~40% continue to suffer from the limitation of moderate intensity exercise (30). Exercise limitation 12 months after PEA is characterized by multifactorial etiologies involving lower RVEF, CI and pulmonary function test abnormalities (FVC, FEV1/FVC, and single breath carbon monoxide diffusing capacity).

### Exercise Training on the Exercise Capacity and QoL

As the primary endpoint, the 6 MWD, peakVO_2_ or peakVO_2_/kg demonstrated significant increases in many studies (11, 19, 28, 29, 31). In our study, we observed significant improvements in exercise tolerance, shown as the 6 MWD, peak VO_2_ or peak VO_2_/kg, after exercise training.

Exercise training performed in different etiologies of PH improved not only the exercise capacity but also different aspects of the QoL, as shown in several studies (16–20, 27, 29).

From the ERS statement of summarized outcomes of the QoL in many studies (27), bodily pain and general health perception always have no significant differences. Bodily pain, general health perception and social role functioning were significantly improved in our study. Our study may indicate that exercise rehabilitation after PEA may be more effective in improving bodily pain, general health perception and social role functioning.

In addition, our study found that exercise training can improve the exercise capacity independent of the effect of the PEA surgery response.

In this systematic review, only Christian Nagel's study described the time interval between the PEA and the initiation of the exercise programme (22). Patients underwent exercise training 3.3 ± 0.9 (median 3.1) weeks after PEA. Other studies did not describe the time interval. There is little information about the acute and chronic effects of PEA on the exercise capacity and ventilatory efficiency in patients with CTEPH. N Nagaya's study examined the changes in exercise training and ventilatory efficiency as indicated by the peak VO_2_ and the VE/VCO_2_ slope after PEA (32). After PEA, the VE/VCO_2_ slope decreased greatly from baseline (before surgery) to the early phase (1 month) and reached a steady level thereafter. In contrast, they noted a continued increase in the peakVO_2_ from the early to the late phase (4 months) after surgery as well as from the baseline to the early phase (32). Surprisingly, the increase in the peak VO_2_ after surgery did not correlate with the decrease in the PVR. The peak VO_2_ is influenced not only by CO during exercise but also by oxygen extraction in skeletal muscles and vasodilatation of the nutrient arterioles within working skeletal muscles (33). From another point of view, exercise training can improve skeletal muscle oxygen extraction and can continue to improve exercise tolerance in CTEPH patients independent of decreased PVR after PEA.

### Hemodynamics, Echocardiography, and Cardiac Function

Thus far, most exercise training trials published in PH have focused on changes in the exercise capacity. Only one prospective, randomized, controlled trial was designed to systematically evaluate changes during rest and exercise by the invasive measurement of hemodynamics as secondary endpoints (31). Altogether, the study revealed significant increases in CI at rest or during peak exercise and decreases in mPAP and PVR at rest in the training group among 73 PAH or inoperable CTEPH patients. In Nicolino Ambrosino's study (21), CTEPH patients were divided into two groups, with and without “good” surgery hemodynamic response. We integrated the data of the two groups into one group before PEA, after PEA and after exercise training. Among the 110 CTEPH patients, they had decreased mPAP, PCWP, TPG, SVR, PVR, and TPR after PEA surgery. PEA surgery increased the CO, CI, and RVEF, respectively. After PEA, CTEPH patients continued exercise training and underwent evaluation at the 3-month follow-up. Compared with before training but after PEA, exercise training can increase the RVEF (Δ3.53%) but not other hemodynamics, such as the RAP, mPAP, PCWP, TPG, CO, CI, SVR, PVR, and TPR.

Most exercise training studies involved performing echocardiography to estimate sPAP and right ventricular functional variables (20). Although not all individual studies revealed significant improvements in echocardiographic parameters (31), the pooled analysis showed that exercise training had a significantly decreased resting sPAP of 3.7 mmHg. Our pooled analysis showed sPAP was assessed by echocardiography at 3 weeks or 12/15 weeks regardless of the resting sPAP or peak sPAP. In our study, we did not pool the analysis of right heart areas because only Christian Nagel's study assessed this outcome (22).

With regard to the cardiac function assessed by CPET (17, 22), the oxygen pulse had a trend toward an increase at 3 weeks. However, regardless of whether at rest or max, neither the HR nor oxygen saturation changed. Meanwhile, NT-proBNP plasma levels continued to decrease with 12 or 15 weeks of exercise training after PEA.

Interestingly, our pooled analysis showed the arterial blood gases at the end of the post-PEA 3-month exercise training significantly improved, similar to the improvement observed at the end of the PEA.

### Limitations

Our study had several limitations. First, we found only a few studies that had assessed the safety and efficacy of exercise training in CTEPH patients after PEA. Studies of early rehabilitation after PEA are scarce, and our study may provide some insights into the safety, tolerability and clinical effects of CTEPH undertaking early exercise training programme after PEA. Second, most studies have not evaluated clinical endpoints, such as right heart failure and mortality. Therefore, we also cannot analyse the impact of exercise training on clinical endpoints. Third, it is difficult to assess the sustainability of the effects of exercise training interventions among CTEPH patients after PEA. Fourth, all included studies were single center and had a shorter duration of follow-up. In the future, multicentre randomized controlled trials with longer follow-up durations are needed to further verify the benefits of exercise training on CTEPH patients after PEA in the real world. Finally, as with all meta-analyses, selection bias cannot be completely excluded. Publication bias could not be assessed because of the insufficient number of research articles.

## Conclusions

The findings of this systematic review and meta-analysis suggest that exercise training may be associated with a significant improvement in the exercise capacity and QoL among CTEPH patients after PEA and proved to be safe. Exercise training also improves arterial blood gases and NT-proBNP. However, additional large-scale and multicentre studies are needed to better evaluate the long-term effectiveness and safety of exercise training in CTEPH after PEA.

## Data Availability Statement

The raw data supporting the conclusions of this article will be made available by the authors, without undue reservation.

## Author Contributions

LW, PY, and RJ contributed to conception and design of the study. Y-LZ, Q-HZ, and RZ organized the database. JH, S-GG, and C-JL performed the statistical analysis. H-LQ wrote the first draft of the manuscript. J-ML, Y-LZ, and PY wrote sections of the manuscript. All authors contributed to manuscript revision, read, and approved the submitted version.

## Conflict of Interest

The authors declare that the research was conducted in the absence of any commercial or financial relationships that could be construed as a potential conflict of interest.
