# Supplementary material for: Comparative Effectiveness of Exercise Training for Patients With Chronic Thromboembolic Pulmonary Hypertension After Pulmonary Endarterectomy: A Systematic Review and Meta-Analysis
Source: Front Cardiovasc Med. 2021 Jun 17;8:664984. doi: 10.3389/fcvm.2021.664984 (PMC8245692; doi:10.3389/fcvm.2021.664984)
Supplement: Supplementary file 2 [file Table_2.DOCX]

Supplementary table2: The National Institutes of Health (NIH) quality assessment tool for before-after (Pre-Post) study with no control group

| Major Components | Response options | | | | |
| --- | --- | --- | --- | --- | --- |
|  | Ekkehard Grüng 2012 | Nicolino Ambrosino 2018 | Christian Nagel 2020 | Takeshi Inagaki 2014 | Joanna Pepke-Zaba  2008 |
| 1. Was the study question or objective clearly stated? | Yes | Yes | Yes | Yes | Yes |
| 2. Were eligibility/selection criteria for the study population prespecified and clearly described? | Yes | Yes | Yes | Yes | Yes |
| 3. Were the participants in the study representative of those who would be eligible for the test/ service/ intervention in the general or clinical population of interest? | Yes | Yes | Yes | Yes | Yes |
| 4. Were all eligible participants that met the prespecified entry criteria enrolled? | Yes | Yes | Yes | Yes | Yes |
| 5. Was the sample size sufficiently large to provide confidence in the findings? | Yes | Yes | Yes | Yes | Yes |
| 6. Was the test/service/intervention clearly described and delivered consistently across the study population? | Yes | Yes | Yes | Yes | Yes |
| 7. Were the outcome measures prespecified, clearly defined, valid, reliable, and assessed consistently across all study participants? | Yes | Yes | Yes | Yes | Yes |
| 8. Were the people assessing the outcomes blinded to the participants' exposures/interventions? | Not Applicable | Not Applicable | Not Applicable | Not Applicable | Not Applicable |
| 9. Was the loss to follow-up after baseline 20% or less? Were those lost to follow-up accounted for in the analysis? | Yes | Yes | Yes | Yes | Yes |
| 10. Did the statistical methods examine changes in outcome measures from before to after the intervention? Were statistical tests done that provided p values for the pre-to-post changes? | Yes | Yes | Yes | Yes | Yes |
| 11. Were outcome measures of interest taken multiple times before the intervention and multiple times after the intervention (i.e., did they use an interrupted time-series design)? | Not Applicable | Not Applicable | Not Applicable | Not Applicable | Not Applicable |
| 12. If the intervention was conducted at a group level (e.g., a whole hospital, a community, etc.) did the statistical analysis take into account the use of individual-level data to determine effects at the group level? | Not Reported | Not Reported | Not Reported | Not Reported | Not Reported |
| **Quality Rating** | Good | Good | Good | Good | Good |
