# Supplementary material for: Comparative Effectiveness of Exercise Training for Patients With Chronic Thromboembolic Pulmonary Hypertension After Pulmonary Endarterectomy: A Systematic Review and Meta-Analysis
Source: Front Cardiovasc Med. 2021 Jun 17;8:664984. doi: 10.3389/fcvm.2021.664984 (PMC8245692; doi:10.3389/fcvm.2021.664984)
Supplement: Supplementary file 3 [file Table_3.DOCX]

| Supplementary table 3 The effect of cardiopulmonary exercise testing on patients with CTEPH | | | | |
| --- | --- | --- | --- | --- |
| Variable | | WMD (95% CI) | % Weight | P-value |
| *Exercise tolerance* | |  |  |  |
| Changes of Peak VO_2_/kg after 3 weeks | | 1.99 (1.48, 2.50) | 16.26 |  |
| Changes of Peak VO_2_/kg after 12/15 weeks | | 4.37 (3.48, 5.26) | 16.17 |  |
|  | | 3.15 (0.82, 5.48) | 53.78 | 0.008 |
| Changes of Peak VO_2_ after 3 weeks | | 158.50 (122.50,194.50) | 1.08 |  |
| Changes of Peak VO_2_ after 12/15 weeks | | 432.09 (349.76, 514.42) | 0.22 |  |
|  | | 292.69 (24.62, 560.75) | 3.45 | 0.032 |
| Changes of VO_2_ at AT after 3 weeks | | 37.07 (-34.72, 108.86) | 0.29 |  |
| Changes of VO_2_ at AT after 12/15 weeks | | 244.50 (133.71, 355.29) | 0.12 |  |
|  | | 136.32 (-66.78, 339.41) | 1.11 | 0.188 |
| Changes of workload max after 3 weeks | | 18.18 (13.05, 23.31) | 12.69 |  |
| Changes of workload max after 12/15 weeks | | 35.83 (27.53, 44.13) | 9.33 |  |
|  | | 26.69 (9.41, 43.98) | 41.65 | 0.002 |
| *Cardiac function* |  | | | |
| Changes of O_2_ pulse after 3 weeks | | 0.98 (0.48, 1.48) | 11.41 |  |
| Changes of O_2_ pulse after 12/15 weeks | | 2.15 (1.50, 2.80) | 10..97 |  |
|  |  | 1.55 (0.40, 2.70) | 33.09 | 0.008 |
| Changes of HR rest after 3 weeks | | -0.36 (-3.16, 2.44) | 4.09 |  |
| Changes of HR rest after 12/15 weeks | | -5.54 (-9.76, -1.32) | 2.21 |  |
|  |  | -2.70 (-7.75, 2.35) | 10.8 | 0.295 |
| Changes of HR max after 3 weeks | | 4.95 (-0.20, 10.10) | 1.58 |  |
| Changes of HR max after12/15 weeks | | 16.25 (9.69, 22.81) | 1.03 |  |
|  |  | 10.41 (-0.66, 21.48) | 4.66 | 0.065 |
| Changes of SaO_2_ rest after 3 weeks | | 0.39 (-0.24, 1.02) | 11.02 |  |
| Changes of SaO_2_ rest after 12/15 weeks | | 0.94 (-0.05, 1.93) | 9.72 |  |
|  |  | 0.55 (0.02, 1.08) | 31.10 | 0.043 |
| Changes of SaO_2_ max after 3 weeks | | 0.57 (-1.31, 2.45) | 6.41 |  |
| Changes of SaO_2_ max after 12/15weeks | | 1.85 (-0.11, 3.81) | 6.16 |  |
|  |  | 1.18 (-0.18, 2.54) | 20.35 | 0.088 |
| SaO_2_: oxygen saturation; PEA: pulmonary endarterectomy; WMD: weighted mean difference. | | | | |
